# A Review of the Medical Testimony, in the Case of Charles B. Huntington

**Published:** 1857-07

**Authors:** Charels F. J. Lehlbach

**Affiliations:** Newark, N. J.


### *A Review of the Medical Testimony in the Case of Charles B. Huntington.* By Charels F. J. Lehlbach, M. D., Newark, N. J.

It is an unfortunate circumstance, that men who strive toward reform —who nobly stand up in defence of the demands and wants of a progressing age—who defy the censures of prejudiced minds, when duty calls to oppose errors, though sanctified by age, are always more ridiculed, sneered at and maligned by the members of their own profession, by the men who, before all, should come to the rescue when great principles and great truths are at stake. It is an unfortunate circumstance; but it must be borne. There is no remedy for it as long as mediocrity has its sway, and this will always be the case. It is so with all prefessions, throughout all classes of men, through all ages. Never was a man arraigned before the public, for the crime of attempting reform, but his professional brethren were the first to throw the stone at him. Never did a man promulgate reformatory ideas, but there arose in his own profession men to question his motives, and if this failed, to try those precious tricks of ridicule and sarcasm, to make light of the matter and thus create in the public mind the impression, as if the reformer was a clever, but half crazy individual, more to be pitied than opposed.

The case of Charles B. Huntington, in whose trial for forgery the plea of insanity was set up—and in our opinion there never was a case, in which this plea was sustained by better arguments, reasons, and facts—is not only an especially interesting and instructive case, but one of great importance. Hence the public and the profession are indebted to the gentlemen, both medical and legal, who have undertaken the task of publishing all the matter connected with it, in the volume whose title appears at the head of this article. Whilst we shall refer the critical reader to this volume, we shall proceed to give a short *résumé* of the bearings of this case for the benefit of those to whom the work is not accessible, and more especially for the benefit of those of our brethren, who are so very loud in their declamations against Drs. Parker and Gilman, and so very anxious to pronounce them "entrapped fools of legal cunning" or "medical heretics." There are men, who, being themselves of excessively small proportions, have a truly insane notion of magnifying their own size by ridiculing those who excel them. We recollect the story of a little, shrimpy-looking dwarf of a doctor, who made great pretensions to obstetric learning, and imagined that the only obstacle in his way to professional eminence was the established professor of that chair in a certain school. Having tried all means to have him leave the faculty, or the faculty leave him, by "*coup de main*" or "*coup de pied*," and having failed, one day a wag told him that the professor had been most shamefully imposed upon by a man, who had sold him a horse for a mare. This was a hoax. But our little friend ran around, everywhere telling people what a shame it was, that a professor of obstetrics should buy a horse for a mare, until he met the professor himself and accosted him: "Doctor, is it true that you have been buying a horse for a mare?"—"No," thundered the professor, "but I have just heard the voice of Balaam's ass." With these remarks, and suggesting to the reader the propriety of keeping his temper, if the opinions here advanced should not agree with his own, we will proceed to discuss the bearings of the medical tessimony in Huntington's case: 1. In regard to the subject of insanity. 2. In regard to its moral aspect. 3. In regard to criminal legislation.

1. The opinions given on the witness-stand by Drs. Parker and Gilman are twofold in their nature and character. They are such as relate to the *general* subject of insanity, while another series of answers, to the questions proposed by counsel, relate to the *special* case of Huntington's insanity. To the philosophical mind these general points in the testimony are of more importance and interest than the secondary question of Huntington's particular case. Instead of Huntington, it might have been Smith or Brown; instead of forgery, the crime might have been homicide or arson; this would have changed the special nature of the case. The accused might have been a woman; this would have turned the sympathies of the public, and even perhaps of the cold-blooded "Times"—that exponent of the philosophy of expediency—in the prisoner's favor; more than this, a high-minded reform-loving judge might have presided over the case and delivered his charge to a more enlightened, more humane, less expediency-philosophizing jury; all these things together would have changed the special nature of the case, but they would not have altered the importance or interest of the testimony given in the case, upon the general question of insanity. These points, however, are sadly overlooked by the public and the profession. Amid the clamors of an excited criticism, even "Gotham, Jr." is led astray and excitingly exclaims: "Who is a criminal if Huntington is insane!"—while he loses sight altogether of the very interesting, very important and most beautiful points in the testimony in regard to the general subject of insanity, which we will presently point out. And these *general* principles of insanity were made the very points of attack by prosecution and judge. Huntington was committed; but not on the ground that the medical testimony was not sufficiently strong to establish his insanity, not on the ground that the physicians were incapable of conducting the examination; nothing of all this, but he was condemned because Judge Capron would not recognize the *principles* of insanity, as laid down by the medical witnesses. We say "Judge Capron:"—if we had said "the law would not recognize them," we would have ascribed to an unknown entity what is justly due to the individual cleverness, legal learning, and conservative wisdom of that honorable gentleman, who has since won still greater laurels. And it is also not so much the alleged fallacy of the opinions of Drs. Parker and Gilman as diagnosticians against which the censures of the medical men are directed, but against their opinions on insanity in general, and especially moral insanity. Thus doctors, lawyers, divines, teachers and Wall Street brokers, all unite to frighten away this ghost of moral insanity, which sprung upon their vivid imagination with all the horrors of an innovation. A little reflection, a little of that kind of investigation which looks at all things, even at forgery, *sine ira et studio*, would convince them that what appears an horrid resolution is but a necessary and wholesome reform, what seems an absurdity is but an old truth, and would show them especially, that there is something very rotten in the present principles of law in regard to insanity, as well as in the practice of criminal legislation. But before analyzing this legal carcass, let us look at the points in the testimony alluded to.

The first point which we will mention, and the most important and interesting, is the clear and triumphant exposition by Drs. Parker and Gilman of the great physiological principle, *that there is no disease of the mind without disease of the body; that insanity implies a morbid condition and an abnormal action of the brain.* When we say "triumphant," we mean that they established this principle before court and jury, in spite of the shrewd, slippery, and adroit cunning of the prosecuting attorney. There are more than half a dozen questions put by that gentleman, the sole object of which, if they had any, was to throw Dr. Parker off his guard, and make him say, that there might be this or the other form of insanity without a change in the nervous centres. The reader may judge for himself by studying the following example of medical firmness unshaken, and legal perseverance under difficulties:—

*Dr. Parker Cross-examined by Mr. Noyes.*

*Q.* Do you mean to say that this *mental organization*[1] was such that he could not resist the impulse or tendency to commit forgery?

*A.* I do: the "tendency," that is the word, sir.

[1] Italics our own.

*Q*. That is the point, then, upon which you placed it—that his *mental* organization was such that he could not resist the tendency to commit forgery? Now, why could he not resist it?

*A*. Because of his diseased organization. *I do not mean mental organization.* The organization of the mind I know nothing about.

*Q*. Do you say physical or mental?

*A*. I say *physical.*

*Q*. You say so, because of his physical organization?

*A*. Yes.

*Q*. So, then, it is his physical organization, when his mental operations are all right?

*A*. I think his mental operations are not right. *They would be if his physical organization was.*

*Q*. They are bad, because his physical organization is bad?

*A*. Yes, because his physical organization is bad.

*Examined by Mr. Brady.*

*Q*. As I understand you, Dr. Parker, all insanity of which you speak, you refer to physical causes?

*A*. The insanity of which we spoke to-day, I refer to physical causes.

*Q*. What we call intellectual insanity applies to the mental process alone?

*A*. The process of the intellect.

*Q*. And moral insanity is as much *a disease of the brain* as the other, but manifests itself in the will, the feelings, and affections, and not in the mental or intellectual process?

*A*. Yes.

*Dr. Gilman Cross-examined by Mr. Noyes.*

*Q*. I find your definition of insanity in an address published, as the best definition you have been able to make: "Insanity is a disease of the brain by which the freedom of will is impaired.

*A*. I admit that sir.

*Q*. I ask you in what respect the freedom of Huntington's will was impaired?

*A*. I suppose Huntington did these things, when he knew they were wrong, *in consequence of his having a disease of the brain.*

*Q*. He had will enough to do them. In what respect was his freedom impaired?

*A*. The temptation was such, that his power of resistance, diminished by *physical organization,* could not overcome it.

*Q*. In other words, he could not resist the temptation from his physical organization?

*A*. From mental defects, dependent on *physical* organization.

This is exceedingly important testimony, and still more so because it stands unrefuted, untouched by the public prosecutor in his summing up, or by the Judge's charge. For years, every intelligent, unprejudiced physician has known this principle to be as correct and true as anything human can be; for years, medical and physiological writers have spoken of it as a thing almost self-evident. And still, when we look over medical testimony of recent cases, where the plea of insanity is set forth, curious to relate, we find persons acquitted and persons condemned on testimony which ignores and denies this principle altogether, testimony which would have passed at par in the last century, but which at the present day is simply ridiculous. About six years ago, a young woman stood accused in our city for the crime of murder.[1] She had, in a fit of passion, stabbed her former paramour, who had been married to another girl, and was acquitted on the plea of insanity. The reader will judge for himself whether looseness of expression and definition, or the ignoring of well established physiological principles is more to be admired in the portion of testimony following.

*Dr. John S. Darcy*, sworn: . . . . I have met with many persons of defective mind; they are frequently cases of temporary derangement from violent causes; great mental excitement has a tendency to impair the intellect of individuals; I have known persons lose the *balance of their minds*[2] from sudden mental excitement [true; but were not such persons predisposed by bodily disease, and was not that sudden mental excitement which made them lose their *balance*, rather the result of previous diseased organization, than merely a mental phenomenon?]; *the loss of reason gives strength to the nerves.* [This is interesting in two points; that the loss of reason gives strength to the nerves may be *new* to many readers, and if absence of reason can strengthen the nerves, then we should be able, by strengthening the nerves, to produce a loss of reason—important in a therapeutical point of view. The only explanation of the phenomenon to which the doctor alludes, that insane persons sometimes make great bodily exertions, is by accepting that the diseased portion of brain which presides over the reasoning faculty communicates its diseased action to that portion of the brain which presides over volition.] . . . . Loss of money or property is very frequently the cause of *mental derangement*, and also the loss of character. [We would beg leave to suggest that the cases of insanity following loss of money or character, are *very* few indeed in proportion to the number of persons who lose both money and character and remain sane, and that hence, in cases where insanity follows such a loss immediately, it has previously existed in a latent form.]

*Gov. Pennington* (counsel for defence): Suppose a female abont 19 should be seduced by a lover; should believe herself pregnant; should be abandoned; continually weep; threaten to destroy herself; dwell continually upon this subject; should be excited; the same lover to marry another person; be found around the house in which they were; is there enough in this case to produce aberration?

*Witness:* I should think in a violent case of that character, where self-destruction was meditated, that it must be a strong intellect indeed, which would so keep its balance, as to be free from a certain state of derangement or *frenzy* of mind, arising, NOT FROM BODILY DISEASE, but from mental excitement. . . . . It is very difficult to determine in cases of great mental disease what is done *with* reason, and what is done without reason. [While we say that the insane has a reason for everything, he does as well as a sane man.] . . . . . . An insane person would be likely to pursue any person who had anything to do with bringing about this state of mind. [Not qnite so fast, doctor; this is not at all a general rule; the religious insane, who form a considerable bulk of the in-

---

[1] The case alluded to is that Margaret Garrity for the murder of Edward Drum. We quote from the full reports of the *Newark Daily Mercury*, October 11, 1851.

[2] Italics our own.

sane, in and out of the lunatic asylums, are generally *rather* attached to those who were more directly concerned in bringing about this state of the mind.] . . . . .

*Mr. Whitehead* (counsel for defence), after relating all the particular circumstances attending the prisoner's conduct during the two weeks previous to the occurrence, and immediately afterwards, as it had been produced in evidence before the court, asked the witness: What was your opinion of her insanity?

*Witness:* I should think, that under all these circumstances, she must have been laboring under *something morbid* (the reader will please to remember, that the doctor does not mean bodily disease), either temporary mental derangement or such a *violent frenzied state of mind* as ought to make her free from all responsibility for her acts on that particular night.

In adducing this testimony, it was not our object to raise the question of Margaret's insanity, though that might be done with impunity, but we only wished to contrast its looseness, want of precision, and lack of scientific foundation with the intelligent, clear, precise, and scientific points of the medical testimony in Huntington's case. Had the prisoner in this case been a man instead of a woman, and the sympathies of the public, the court, jury, counsel, and *prosecution* been against the prisoner instead of in her in favor, this testimony would have been received for what it was worth, and treated with well-deserved ridicule, to say the least of it. But is the public to blame; are judges and jurymen to blame; if "fancy run mad" is received as ocular wisdom, when the accused is a female and the crime murder, while "truth transparent" is received as flimsy theory, when the prisoner is a male, and the crime forgery? No, we need not arraign them for such perversions of justice; the cause lays at home, at the doors of our own profession. As long as the profession silently permit medical witnesses to spread opinions broadcast over the land, which ignore and defy the present state of science; if statements opposed to established facts, as well as to sound sense, are permitted to go as evidence without even an attempt toward a protest from the profession; so long must the life, liberty, and peace of the innocently accused or the punishment of the alleged insane, depend upon a mere lottery of circumstantial good luck, instead of upon truth and justice eternal.

We have said, that this point of materiality of insanity is an important one. It is in fact the only platform upon which the physician as such, can logically appear as a witness in insanity. Deny that insanity is a disease of the brain, and what more qualification has the physician to analyze the phenomena of insanity, than the philosopher, divine, or lawyer? Ay, less! The metaphysician ought to have a better insight into the *mere mental* operations than the physician; the lawyer ought to have as sharp an eye to view mere mental phenomena, as the physician, and the divine whose avocation is based entirely upon the spiritual nature of man should be the best judge of all. What then is the distinctive mark between the physician and the rest? It is this—the philosopher studies little their causes, while he investigates mental processes; the lawyer and divine both analyze the manifestations of the mind and soul, but they heed not that a blow upon the head, a ruptured vessel in the brain, softening of its hemispheres, causes the manifestations of the mind to change or to cease altogether. When the plea of insanity is set forth, society does not wish to know whether a man is capable to reason according to the laws of logic; if so, the metaphysician would be called upon the stand: society does not wish to know whether the alleged insane is sound on dogmata or articles of faith, else the clergyman would be the person to give an opinion; but society wishes to know, *whether the physical organization of a man is such, that he is able, or was able at a given time, to exercise his will in a state of freedom: and to testify upon any other basis, the physician should always refuse.*

The physician knows, that if a certain portion of the brain is removed, though the ganglia of the various senses remain, all power of intelligent volition is lost. He also knows, that if the portion of brain in which the will resides be affected with disease, the manifestations of the will must be abnormally modified according to the nature and severity of the disease; he knows that in the vast majority of cases of insanity the connection between the mental manifestations and their causes can be directly traced to a diseased condition of the brain; and he knows that a perversion of the senses, of the will or intellect, which annuls the freedom of will, can only be produced by one of two things; either by disease of the brain or some other portion of the nervous system, or by a miracle; miracles, however, are not taken in evidence before court and jury, though the time was, and is to a certain extent yet, when the ignorance of physicians, or the superficiality of their post-mortem examinations was covered with the solemn verdict of "a visitation of providence." Judges and prosecuting attorneys are very quick in pronouncing medical testimony on insanity as mere theory, sheer hypothesis, flimsy supposition, and by way of comparison they are fond of alluding to the "well settled" principles of law in regard to insanity, which have stood "the test of time." This "test of time," however, reminds us of those corpses, which when embalmed and preserved in air-tight coffins, stand the test of many centuries, but as soon as they are brought to light, crumble into dust. The lawyer assures us, and so does the judge, that it is a flimsy theory when the physician asserts, that insanity is a disease of the brain. But, sir, this theory, if so you choose to call it, is supported by an immense accumulation of facts and experience. "I have nothing to do with your facts and experience, the principles of law have stood the test of time." What shall medical men answer to such obstinacy? Is a theory correct and true because it has existed for a long time? Was heathenism true because it had the "test of time" for many centuries, until Christianity conquered its deities? No! the very allusion of those who deny the materiality of insanity to the old age and venerability of their unsupported theories and assertions, should fill us with suspicion. For the last reason which always induces men to defend false theories, erroneous principles and bad institutions, is their very age and venerability. We may close this part of our notice with the simple statement, that so far as we have been able to find, the case of Huntington is the first in the United States, where the medical witnesses asserted and established the materiality of insanity, as a scientific principle, and thereby also established the legitimacy of medical testimony in insanity.

The second point of importance in the testimony of Drs. Parker and Gilman, is the question of moral insanity. It may at first sight appear somewhat strange, that the very men who hold that insanity means disease of the brain, should accept the existence of such a thing as moral insanity, because we often speak of the moral nature of men as opposed to his physical nature. But the apparent contradiction is easily solved when we look, not at the mere term, but at the thing which the term is meant to imply. By moral insanity is understood simply a morbid state of the brain, excluding the freedom of will without an apparent disturbance of intellect; a condition of the brain by which a man is *forced* to an act, though he know that act to be wrong. The cases adduced by Dr. Gilman in his "medico-legal examination of the case of Ch. B. Huntington" established this point beyond the shadow of a doubt—not as a theory, but as a positive fact, and we earnestly solicit every member of the medical, as well as the legal profession, to possess himself of the pamphlet, and dare after his perusal to pronounce moral insanity but a fable. Why, this moral insanity is as common as any other form of insanity, only not so generally recognized, because it more frequently manifests itself in small matters and minor wrongs, which do not become the subject of judicial interference or criminal prosecution. There are men who pertinently tell lies; they are aware of the wrong they commit in doing so; aware of the consequences, but still they will lie; lie a man out of his own existence if they can. The mode in which this moral insanity manifested by lying is brought on, can often be traced to the habit acquired in early childhood, to amuse or being amused by "strong" stories. This habit becomes finally so interwoven into a man's very existence, and, for aught we know, into his brain, that he cannot, if he would, resist the impulse to lie. What was sane immorality at first, becomes moral insanity at the end. A man may, by repeatedly doing a certain act, produce such a condition of his brain, that this very condition will force him to repeat the act again, and again, though he is perfectly well aware of the wickedness of the act and its consequences. Poor Edgar A. Poe; all his poetical and intellectual powers did not prove barriers against that horrible affliction, moral insanity, which creeps into the brain unfelt, unseen, makes man a brute, he knows not how, makes him a devil, he knows not why.

Still, another series of cases in the history of crime, showing that insanity *can* exsit without loss of intellect, and with the knowledge of right and wrong, are cases of suicide. Self-preservation is the first law throughout the living world, and disease only can destroy that innate desire in man, and supplant it by the desire of self-destruction. Who will say that a man with a sound body and a healthy brain, whose freedom of will is thus unimpaired, will meditate and commit suicide? The very idea is preposterous. But while those who commit suicide are insane, as insane as man can possibly be, have they not, except when the deed is done in the wild delirium of an acute disease, full power of reasoning, full knowledge of right and wrong? Is it necessery to quote the letters of such unfortunate beings, full of intellect, and closing with the words, "May God forgive me!" Does not this clearly show that insanity is compatible with the possession of intellect, and the knowledge of right and wrong? What then are the bearings of the medical testimony in Huntington's case? We answer—

1. It has established the legitimacy of medical testimony in insanity, on a basis of positive science.

2. It has for the first time on this side of the Atlantic brought before court and jury, the *fact* of moral insanity, and thereby

3. Exposed the utter fallacy of the present principles of law, in regard to insanity.

Achievements of which any man, however high he may stand in professional eminence, may justly feel proud.

(Space and time do not permit us to touch the moral and practical points of the question on this occasion.)—*N. J. Medical & Surgical Reporter.*

